# Clinical implications of plasma sADAM10: integrating renal function, histopathology and prognostic outcomes in chronic kidney disease and kidney transplantation

**DOI:** 10.3389/fimmu.2026.1752330

**Published:** 2026-07-02

**Authors:** Xueqiao Wang, Yan Luo, Qu Yang, Hua Zhang, Yunfei An, Xinhua Dai, Yangjuan Bai, Yamei Li

**Affiliations:** 1 Department of Laboratory Medicine/Clinical Laboratory Medicine Research Center, West China Hospital, Sichuan University, Chengdu, Sichuan, China; 2 Sichuan Clinical Research Center for Laboratory Medicine, Chengdu, Sichuan, China; 3 Department of Pathology, General Hospital of Western Theater Command, Chengdu, Sichuan, China; 4 Tissue Stress Injury and Functional Repair Key Laboratory of Sichuan Province, General Hospital of Western Theater Command, Chengdu, Sichuan, China

**Keywords:** biomarker, chronic kidney disease, infection risk, kidney transplantation, prognosis, soluble ADAM10

## Abstract

**Background:**

The identification of novel non-invasive biomarkers is crucial for improving the management of chronic kidney disease (CKD) and kidney transplant recipients (KTR). While A Disintegrin and Metalloprotease 10 (ADAM10) is involved in kidney pathology, the clinical relevance of its soluble form, soluble ADAM10 (sADAM10), remains poorly defined.

**Methods:**

In this retrospective cohort study, we enrolled 488 participants, including 145 kidney transplant recipients (KTR), 295 patients with chronic kidney disease (CKD), and 48 healthy controls (HC). Plasma sADAM10 was measured by ELISA and evaluated for its correlation with renal function, laboratory parameters, and histopathological findings. Its prognostic value for adverse outcomes was determined using Kaplan-Meier analyses, restricted cubic spline (RCS) Cox regression, and incremental value assessment.

**Results:**

Plasma sADAM10 were significantly elevated in biopsy-proven KTR and CKD patients compared to HC. Multivariate analyses revealed cohort-specific independent associations: plasma sADAM10 was independently associated with eGFR in CKD (standardized β = –0.24, P < 0.001), but with neutrophil count in KTR (standardized β = 0.24, P = 0.034). Histopathologically, higher plasma sADAM10 were associated with the severity of interstitial fibrosis and tubular atrophy (IFTA), tubular injury, and interstitial inflammation. RCS analysis demonstrated a significant non-linear association between plasma sADAM10 and post-transplant infection risk (P for non-linearity = 0.029), with plasma sADAM10 providing incremental prognostic value beyond age and eGFR (C-index increase from 0.795 to 0.842; likelihood ratio P = 0.005). In contrast, after adjusting for eGFR and proteinuria, plasma sADAM10 showed no independent association with the composite renal endpoint. For patients with advanced CKD, plasma sADAM10 concentration showed an exponential association with the predicted 5-year risk of kidney failure.

**Conclusion:**

Plasma sADAM10 is a context-dependent biomarker with endpoint-specific prognostic value. It shows an independent association with renal dysfunction in CKD and inflammatory activation in KTR, and predicts post-transplant infection risk with incremental value beyond traditional markers. However, its apparent association with renal outcomes is confounded by eGFR. These findings highlight the potential of plasma sADAM10 for infection risk stratification in KTR and warrant further validation in larger cohorts.

## 1 Introduction

Chronic kidney disease (CKD) poses a significant global public health challenge, associated with substantial morbidity and mortality. In 2017, CKD affected approximately 700 million individuals worldwide and was responsible for 1.2 million deaths attributable to CKD-related disorders (1, 2). This burden is expected to increase further, driven by aging populations and the growing prevalence of risk factors such as hypertension, obesity, and type 2 diabetes mellitus (2, 3). According to the Kidney Disease: Improving Global Outcomes (KDIGO) guidelines, CKD diagnosis relies on the persistence of kidney structural or functional abnormalities, assessed primarily through glomerular filtration rate (GFR) and albuminuria (4). CKD disproportionately affects low- and middle-income countries, where access to early diagnosis and treatment remains limited compared to high-income regions (3, 5). Identifying novel biomarkers for early diagnosis and developing effective interventions are therefore crucial to slow CKD progression and reduce its associated burden. Moreover, patients with CKD are at high risk of progressing to end-stage kidney disease (ESKD), necessitating costly renal replacement therapy, including dialysis or kidney transplantation (KT). Although KT offers better quality of life and long-term survival than long-term dialysis (6), it is complicated by challenges such as allograft rejection and immunosuppression-related complications (7, 8). Current monitoring strategies largely depend on traditional biomarkers like estimated glomerular filtration rate (eGFR) and urine protein, which have limited sensitivity and specificity for detecting early graft injury. Thus, there is a critical need for innovative non-invasive biomarkers to enable early and accurate assessment of graft status and immune risk (9, 10).

A disintegrin and metalloprotease 10 (ADAM10) acts as a key “molecular scissor,” cleaving diverse substrates including Notch and cadherins to regulate essential signaling pathways (11, 12). In the kidney, its expression in tubular cells is critical for maintaining brush border gene expression and epithelial barrier integrity, largely through cleavage of proteins such as E-cadherin (11, 13). ADAM10 is a zinc-dependent transmembrane protease that plays a pivotal role in cellular communication and tissue homeostasis by mediating the ectodomain shedding of membrane-bound substrates such as Notch, epidermal growth factor (EGF), and E-cadherin (14, 15). In the context of kidney diseases, this proteolytic activity links ADAM10 to key pathological processes, including epithelial-mesenchymal transition (EMT), renal fibrosis, and podocyte injury. ADAM10 has been implicated in EMT in tubular epithelial cells, where its upregulation (e.g., via PAX2) promotes E-cadherin cleavage and reduces cell–cell adhesion (16). Beyond its role in EMT, ADAM10 also contributes to fibrosis by shedding Notch receptors and ligands (e.g., Delta), thereby activating the Notch signaling pathway that drives nephritis and fibrosis (17). In podocytes, ADAM10 knockdown attenuates inflammation and apoptosis by suppressing MAPK pathway activation (18), whereas its mediated cleavage of podocyte surface proteins such as THSD7A and PLA2R1 exacerbates injury in MN (17, 19, 20). Apart from its pathogenic roles, ADAM10 also participates in normal kidney development and tubular function maintenance through Notch signaling and N-cadherin shedding (13, 21). Given its central roles in inflammation, fibrosis, and cell differentiation, ADAM10 has garnered increasing interest in the context of CKD and KT (14, 22–24).

Soluble ADAM10 (sADAM10) is a proteolytically active form that lacks the transmembrane and cytoplasmic domains, likely generated through proteolytic cleavage or alternative splicing, although the precise mechanisms remain incompletely understood (25). In our previous study, we observed that reduced ADAM10 expression on monocytes was closely associated with renal allograft dysfunction in KT recipients (KTR) (26). Motivated by the multifaceted roles of ADAM10 in renal pathology and this prior finding, we hypothesized that its soluble form might hold broader clinical relevance. Notably, elevated plasma sADAM10 have been reported in Alzheimer’s disease, supporting its biomarker potential in conditions involving inflammation and ectodomain shedding (27–29), which are also commonly observed in kidney diseases. However, the clinical relevance of sADAM10 and its potential as a biomarker in CKD and allograft dysfunction remain unclear. To address this gap, this study aimed to investigate whether sADAM10 may serve as a useful biomarker for diagnosis, risk stratification, and prognostication in both CKD and KT, by integrating data of plasma sADAM10 with comprehensive assessment of renal function, laboratory parameters, histopathological severity, and longitudinal clinical outcomes.

## 2 Materials and methods

### 2.1 Study population

This was a retrospective cohort study utilizing prospectively collected biospecimens and clinical data. A total of 488 participants were enrolled from West China Hospital of Sichuan University, including 145 KTR, 295 patients with native CKD, and 48 healthy controls (HC). The KTR cohort consisted of adult patients (≥18 years) who had undergone KT; those who had received multi-organ transplants or had active systemic infections at the time of enrollment were excluded. Among the KTR, 31 with stable allograft function (stable group) and 114 who underwent indication allograft biopsy due to suspected rejection, unexplained graft function decline, or proteinuria were finally included. The CKD cohort included adult patients diagnosed according to the KDIGO guidelines (4). To ensure pathological homogeneity, we specifically enrolled 122 patients with biopsy-proven membranous nephropathy (MN) and 173 with biopsy-proven IgA nephropathy (IgAN). Individuals with concomitant systemic diseases, including active infections, malignancies, or other immune disorders, were excluded. The HC group comprised adult volunteers (≥18 years) with no self-reported history of kidney disease, hypertension, diabetes, or other systemic illnesses, and with normal routine laboratory test results. This study was approved by the Institutional Review Board of West China Hospital, and written informed consent was obtained from all participants. 

### 2.2 Sample collection and plasma sADAM10 measurement

Peripheral blood samples were collected from all participants prior to renal biopsy. Plasma was separated and stored at –80 °C until analysis. The concentration of plasma sADAM10 was quantified using a commercial human ADAM10 enzyme-linked immunosorbent assay (ELISA) kit (JONLNBIO, Cat. No. JL13337, Shanghai, China), strictly in accordance with the manufacturer’s instructions.

### 2.3 Laboratory and clinical data collection

Hematological and biochemical data were retrieved from the hospital’s laboratory information system. Complete blood counts, including neutrophil and lymphocyte counts, were performed using a Sysmex XN-3100 automated hematology analyzer. Serum biochemical parameters, including renal function, liver function, and lipid profiles, were measured on a Roche cobas c702 clinical chemistry analyzer. Urine protein was semi-quantitatively assessed by dipstick. Renal histopathological data for KTR and CKD (MN and IgAN) patients were obtained from clinical histology reports. Allograft biopsies from KTR were assessed using the Banff 2022 classification (30), while native kidney biopsies from CKD patients were evaluated based on integrated findings from histomorphology, immunofluorescence, and electron microscopy. Pathological lesions were evaluated and graded according to established international criteria. These included glomerulosclerosis, mesangial matrix expansion, crescent formation, tubular epithelial cell (TEC) injury (grades of mild, moderate, or severe directly extracted from descriptive histopathology reports), interstitial fibrosis and tubular atrophy (IFTA), interstitial inflammation, and arteriolar hyalinosis.

### 2.4 Statistical analyses

All statistical analyses were performed using R software (version 4.4.3). Data visualization was conducted with the ggplot2 and ggpubr packages. Continuous variables are expressed as median (interquartile range, IQR), while categorical variables are presented as frequency (percentage). Group comparisons for continuous variables were carried out using non-parametric tests: the Mann–Whitney U test for two-group comparisons and the Kruskal–Wallis test for multi-group comparisons, followed by Dunn’s test for *post-hoc* analysis. Categorical variables were compared using the chi-square test or Fisher’s exact test, as appropriate.

The association between plasma sADAM10 and continuous clinical parameters was assessed using Spearman’s rank correlation coefficient (ρ). A locally weighted scatterplot smoothing (LOESS) regression model, with a smoothing parameter (span) of 0.75, was applied to explore potential nonlinear relationships between plasma sADAM10 and eGFR. This span value was selected to optimally capture underlying trends while minimizing overfitting. For prognostic analysis in the KTR cohort, two primary endpoints were defined (1): time to first post-biopsy infection, and (2) a composite renal endpoint, defined as allograft failure or a ≥30% decline in eGFR. The optimal cutoff value of plasma sADAM10 for stratifying patients into high- and low-risk groups for each endpoint was determined using the maximum selected rank statistics method. Survival curves were plotted using the Kaplan–Meier method and compared with the log-rank test. Univariate Cox proportional hazards models were used to estimate hazard ratios and corresponding 95% confidence intervals. To assess potential non-linear relationships and avoid bias from data-driven cut-off selection, we performed restricted cubic spline (RCS) Cox regression with three knots placed at the 10^th^, 50^th^, and 90^th^ percentiles, modeling plasma sADAM10 as a continuous variable. The 5^th^ percentile of plasma sADAM10 was used as the reference. For the infection endpoint, models were adjusted for age and sex; for the composite renal endpoint, adjustments included age, sex, eGFR, and proteinuria. The Wald test was used to evaluate the overall significance of plasma sADAM10 and its non-linear component. To evaluate whether plasma sADAM10 provides incremental prognostic information beyond traditional risk factors, nested Cox proportional hazards models were compared. For each endpoint, a baseline model including established predictors was constructed. An extended model additionally incorporated plasma sADAM10, either as a linear term or as a restricted cubic spline with three knots to capture potential non-linear effects. Model fit was assessed using the likelihood ratio test, and discrimination was evaluated using Harrell’s C-index derived from the model’s Somers’ Dxy rank correlation. Additionally, in a subgroup of patients with advanced CKD (stages G3–G5), we evaluated the relationship between plasma sADAM10 and the predicted 5-year risk of kidney failure (31). A two-sided P < 0.05 was considered statistically significant for all analyses.

## 3 Results

### 3.1 Baseline characteristics of study participants

The baseline characteristics of all participants are summarized in **Table 1**. Demographically, the KTR cohort was characterized by a predominance of male patients (73.1%) and a lower median age (37.0 years). In contrast, the CKD and HC groups were older and exhibited a more balanced sex distribution. As expected, renal function parameters differed markedly across the groups. KTR demonstrated the most pronounced renal dysfunction, with the lowest median eGFR (43.11 mL/min/1.73m²). Patients in the CKD cohort showed moderate impairment, whereas HCs maintained normal renal function. Proteinuria was highly prevalent in both patient cohorts (KTR: 75.9%; CKD: 96.9%) but absent in the HC group. Among the KTR, the median time from transplantation to blood sampling was 1179 days (range 8–4955 days; IQR 444–2162 days). Spearman correlation analysis revealed no significant association between plasma sADAM10 and post-transplant days (ρ = 0.12, P = 0.24). Other laboratory parameters, including liver enzymes and lymphocyte counts, were comparable across all three groups and remained within normal ranges. Given the heterogeneous composition of the KTR cohort (stable patients vs. those with indication for biopsy), we compared baseline characteristics between the two subgroups (**Supplementary Table 1**). The indication-biopsy group showed significantly elevated plasma sADAM10, worse renal function, and increased inflammatory markers compared with the stable group (all *P* < 0.01).

**Table 1:** Baseline characteristics of study participants.

Variable	KTR	CKD	HC
Number	145	295	48
Gender			
Female	39 (26.9%)	155 (52.5%)	30 (62.5%)
Male	106 (73.1%)	140 (47.5%)	18 (37.5%)
Age(years)	37.00 (31.00-47.00)	41.00 (31.00-54.00)	43.00 (40.00-50.00)
Urine protein			
–	35 (24.1%)	9 (3.1%)	52 (100.0%)
±	14 (9.7%)	14 (4.7%)	–
1+	28 (19.3%)	53 (18.0%)	–
2+	41 (28.3%)	100 (33.9%)	–
3+	21 (14.5%)	63 (21.4%)	–
4+	6 (4.1%)	56 (19.0%)	–
Time after transplantation (days)	1437.00 (717.00-2369.00)	–	–
Tacrolimus concentration (ng/mL)	5.40 (4.56-6.69)	–	–
Body mass index (kg/m²)	21.17(18.80-24.31)	23.19(20.81-25.82)	–
Serum creatinine (μmol/L)	166.00 (103.00-246.00)	82.50 (65.00-109.00)	66.50 (58.75-76.00)
eGFR (mL/min/1.73m²)	43.11 (26.15-73.49)	86.61 (65.00-105.66)	103.78 (94.89-110.34)
Alanine aminotransferase (U/L)	14.00 (9.00-18.00)	16.00 (11.00-24.00)	17.50 (13.00-27.00)
Aspartate aminotransferase (U/L)	16.00 (12.00-19.00)	18.00 (15.00-23.00)	19.50 (16.75-22.25)
Neutrophil count (10^9^/L)	5.73 (4.33-7.38)	4.03 (3.18-5.22)	2.96 (2.40-3.40)
Lymphocyte count (10^9^/L)	1.75 (0.93-2.30)	1.75 (1.41-2.10)	1.65 (1.46-1.96)

Data are presented as median (IQR) or n (%). Formal comparisons between groups were not performed due to inherent differences in these distinct clinical populations; all subsequent analyses were adjusted for relevant confounders within each cohort.

### 3.2 Distribution and clinical correlation of plasma sADAM10

**Figure 1:**
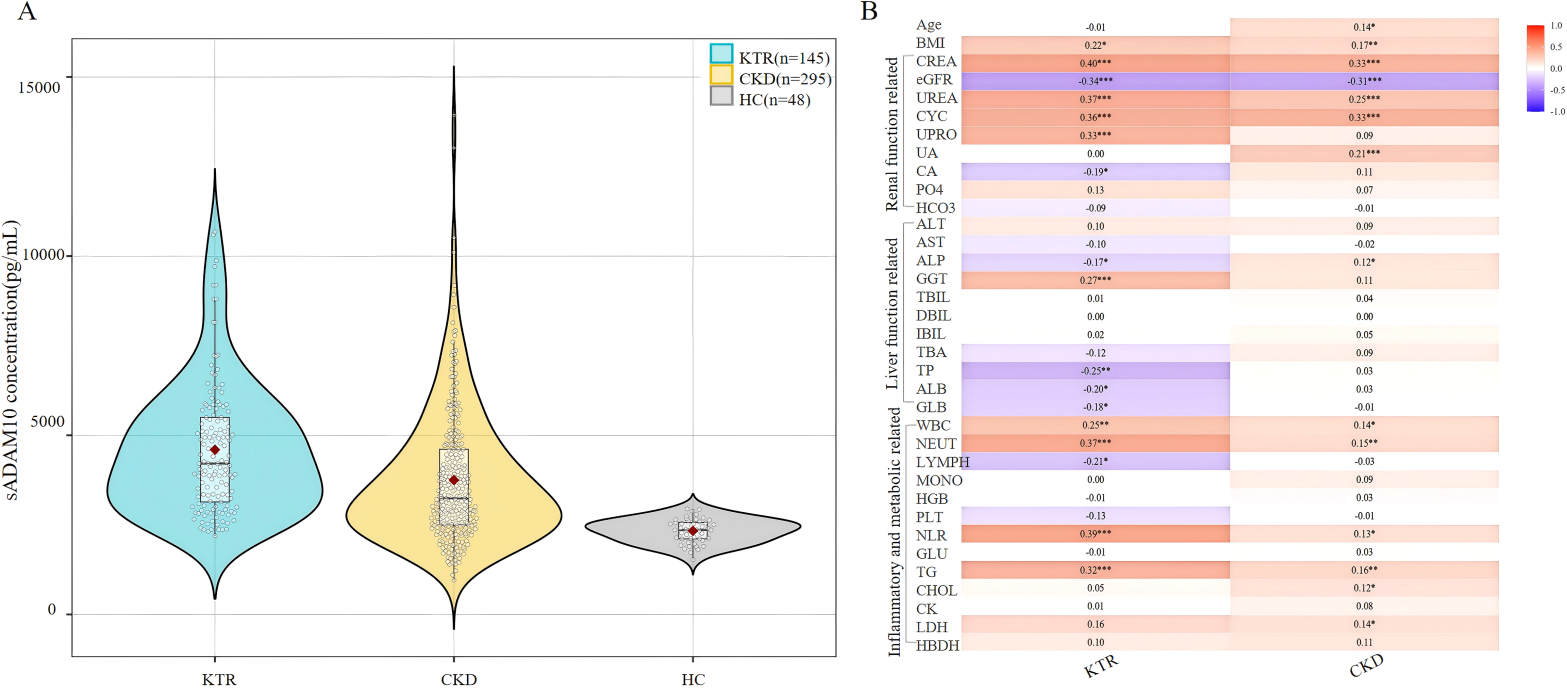
Plasma sADAM10 distribution and correlation analyses between plasma sADAM10 and laboratory parameters in KTR and CKD groups. **(A)** Violin plot illustrating the distribution of plasma sADAM10, the width of the violin represents the probability density (wider sections indicate higher density of data points). Embedded box plots display the median (horizontal line) and interquartile range (box boundaries); whiskers extend to 1.5×IQR. Translucent white dots represent individual measurements, and red diamonds indicate group means; **(B)** Correlation analyses between plasma sADAM10 and clinical parameters. *P<0.05; **P<0.01; ***P<0.001.

As shown in **Figure 1A**, plasma sADAM10 were significantly higher in KTR (median 4210.28 pg/mL, IQR 3139.72–5498.72) and CKD patients (median 3238.67 pg/mL, IQR 2499.03–4611.05) compared with healthy controls (median 2356.16 pg/mL, IQR 2110.32–2565.73) (both *P* < 0.001). The violin plots illustrate that both patient groups exhibited broader, right−skewed distributions relative to the healthy controls. In both patient cohorts, plasma sADAM10 correlated with a range of clinical parameters (**Figure 1B**). Demographically, a weak positive correlation with body mass index (BMI) was observed in both groups. With respect to renal function, plasma sADAM10 consistently demonstrated weak-to-moderate positive correlations with serum creatinine, urea nitrogen, and cystatin C (ρ = 0.25–0.40), along with moderate negative correlations with eGFR (ρ = –0.31 to –0.34). Furthermore, positive correlations were observed with inflammatory markers, such as notably neutrophil count and neutrophil-to-lymphocyte ratio, which were more pronounced in KTR than in the CKD group. Multivariate analyses revealed cohort-specific independent associations: in CKD patients, plasma sADAM10 remained significantly associated with eGFR after full adjustment (standardized β = –0.24, P < 0.001), whereas associations with neutrophil count and NLR were attenuated and became non-significant after including eGFR. In contrast, in KTR, plasma sADAM10 was independently associated with neutrophil count (standardized β = 0.24, P = 0.034) but not with eGFR (**Supplementary Table 2**).

### 3.3 Plasma sADAM10 exhibits a threshold-based nonlinear association with renal function

**Figure 2:**
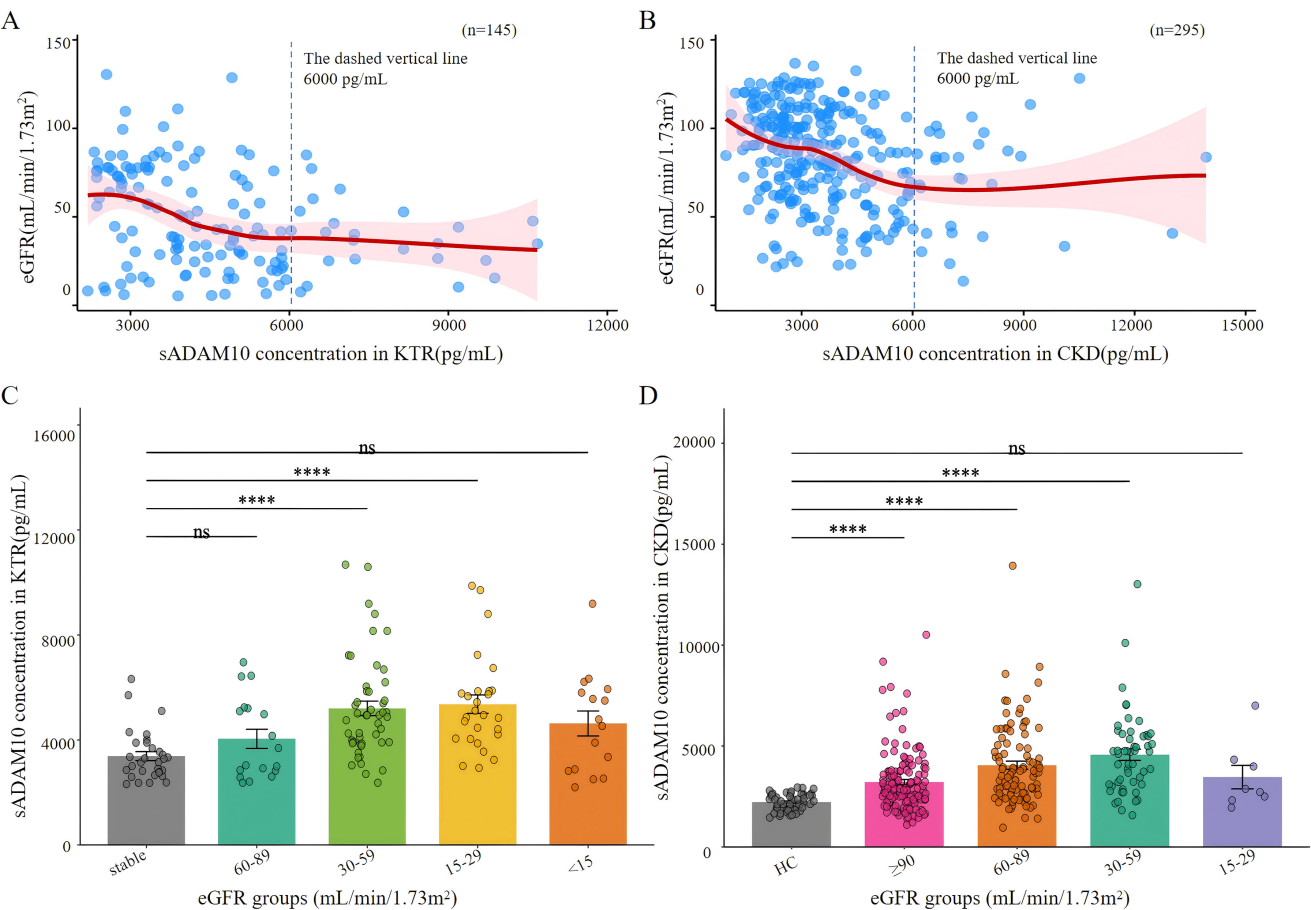
Relationship between plasma sADAM10 and eGFR levels in KTRs and CKDs. **(A)** Loess regression curve illustrating the association between plasma sADAM10 and eGFR in the KTR cohort; **(B)** Loess regression curve illustrating the association between plasma sADAM10 and eGFR in the CKD cohort; **(C)** Bar graph comparing plasma sADAM10 across stratified eGFR groups in KTR; **(D)** Bar graph comparing plasma sADAM10 across stratified eGFR groups in CKD patients. ****P<0.0001.

LOESS regression analyses revealed a consistent nonlinear relationship between plasma sADAM10 and eGFR in both KTR and CKD cohorts (**Figures 2A, B**). A significant negative correlation was observed at plasma sADAM10 below 6000 pg/mL (Spearman’s ρ ≈ –0.3, P < 0.001), beyond which the association plateaued, indicating a threshold effect. This pattern remained evident when patients were stratified by eGFR categories in both cohorts (**Figures 2C, D**). Specifically, plasma sADAM10 were significantly higher in patients with moderate-to-severe renal impairment (eGFR stages 3–4) than in those with preserved renal function. Interestingly, in both KTR and CKD patients with the most advanced kidney disease (eGFR < 30 mL/min/1.73m²), plasma sADAM10 did not differ from those in HC. Furthermore, among patients with advanced CKD (eGFR < 60 mL/min/1.73m²), plasma sADAM10 remained relatively stable across progressively declining eGFR strata.

### 3.4 Plasma sADAM10 associates with fibrosis and inflammation in kidney pathology

To evaluate the relationship between plasma sADAM10 and kidney histopathological features, we initially compared plasma sADAM10 across specific pathological diagnoses in KTR (including antibody‐mediated rejection [ABMR], focal segmental glomerulosclerosis [FSGS], and recurrent or new‐onset IgAN) and in CKD (MN and IgAN). No significant differences in plasma sADAM10 were observed among these diagnostic subgroups (**Supplementary Figure 1**).

**Figure 3:**
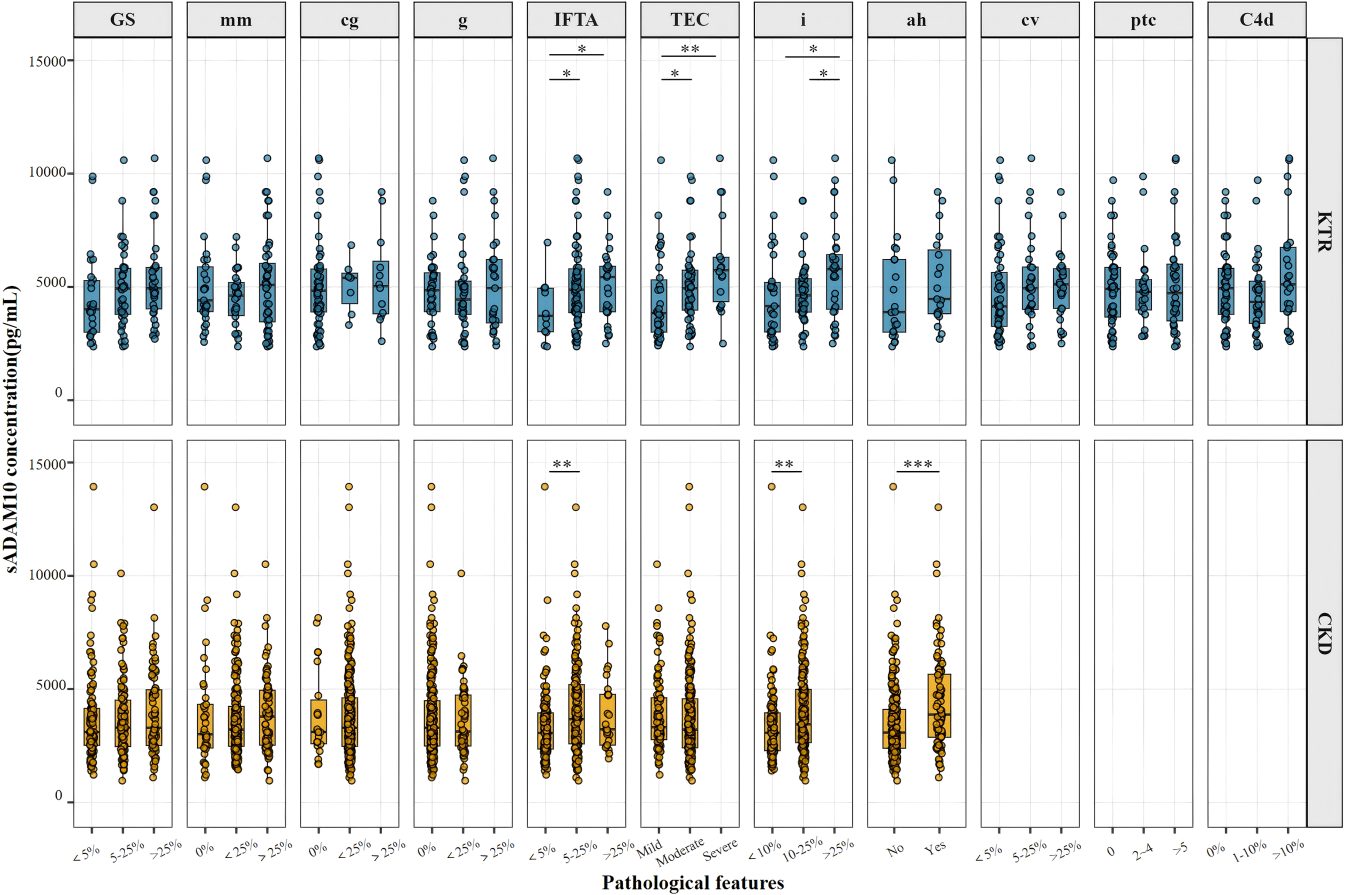
Analysis of plasma sADAM10 concentration and pathological parameters in KTR and CKD patients. *P<0.05; **P<0.01; GS, glomerulosclerosis (% of glomeruli); mm, mesangial matrix expansion (% of glomerular involvement); cg, chronic glomerulopathy (% of glomerular scarring); g, glomerulitis (% of glomerular involvement); IFTA, interstitial fibrosis and tubular atrophy (% of cortical area); i, interstitial inflammation (% of cortical area); cv, vascular fibrous intimal thickening (% of luminal stenosis); C4d, C4d staining (% of peritubular capillary positivity); TEC, tubular epithelial cell injury (graded as: mild, moderate, severe); ah, arteriolar hyalinosis (graded as: present or absent); ptc, peritubular capillaritis (graded as: 0, 2-4, or >5 inflammatory cells per tubule).

Subsequent analyses revealed disease‐specific associations between plasma sADAM10 and the severity of particular histological lesions (**Figure 3**). In KTR, plasma sADAM10 increased significantly with the severity of IFTA, TEC injury, and interstitial inflammation. Specifically, plasma sADAM10 were significantly higher in patients with extensive fibrosis (>25% cortical area) compared to those with mild (5–25%; P = 0.046) or minimal (<5%; P = 0.043) involvement. Severe TEC injury and extensive interstitial inflammation were also associated with markedly elevated plasma sADAM10. When analyzing the native CKD cohort as a whole (combining MN and IgAN), elevated plasma sADAM10 was significantly associated with IFTA (P = 0.004), interstitial inflammation (P = 0.003), and arteriolar hyalinosis (P = 0.0003). No significant associations were observed with glomerulosclerosis, mesangial matrix expansion, or crescent formation. Detailed subgroup comparisons between MN and IgAN are presented in **Supplementary Figure 2**, which reveals disease−specific patterns (e.g., IFTA and interstitial inflammation were significantly associated with plasma sADAM10 in MN but not in IgAN, whereas arteriolar hyalinosis showed associations in both).

### 3.5 Plasma sADAM10 demonstrates prognostic value for risk stratification in KTR and advanced CKD patients

**Figure 4:**
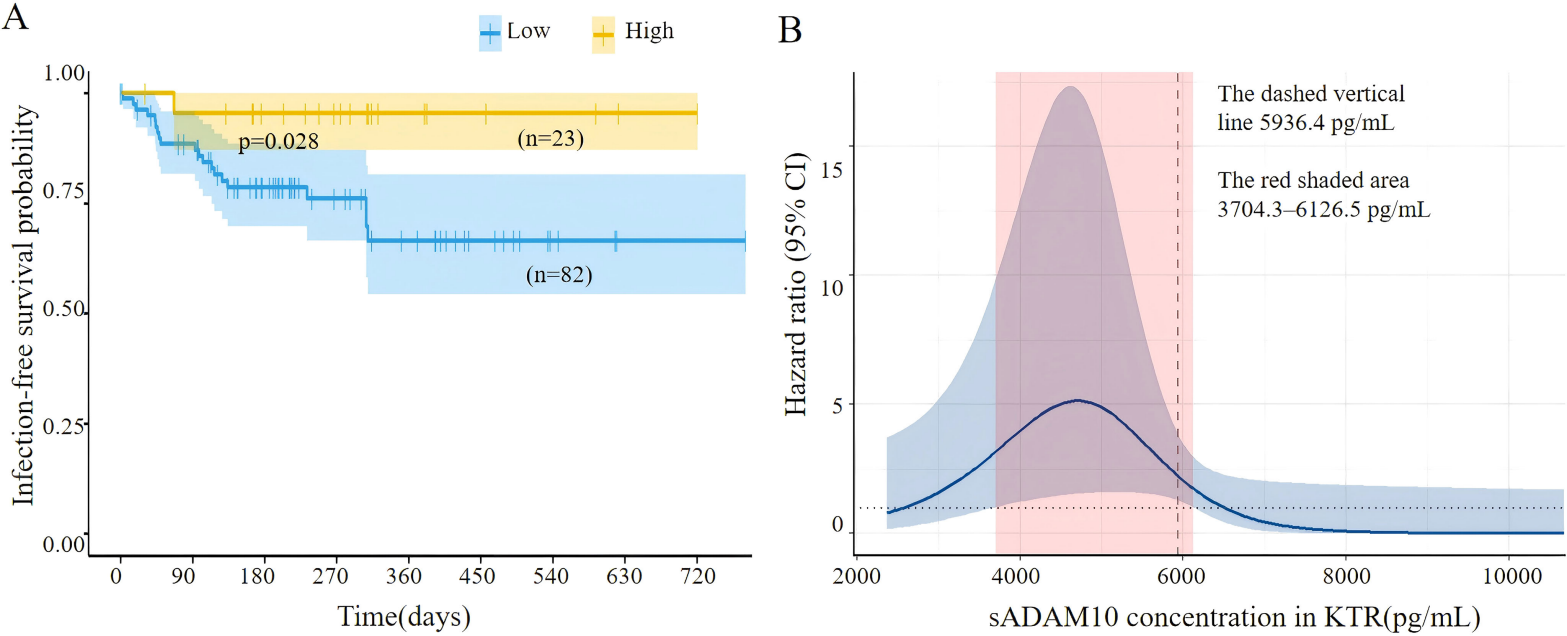
Prognostic value of plasma sADAM10 for predicting adverse outcomes in KTR. **(A)** Kaplan-Meier analysis for infection outcomes in KTR; **(B)** Nonlinear association between plasma sADAM10 and infection risk assess.

Given the marked differences in baseline characteristics between stable patients and those undergoing indication biopsy, together with the very low number of outcome events in the stable group (e.g., only 2 infections), prognostic analyses were restricted to the indication−biopsy subgroup (n=114). Within this subgroup, we found that plasma sADAM10 significantly predicted adverse outcomes in KTR. Using the maximum selected rank statistic method, we stratified patients into high and low plasma sADAM10 groups. For the infection endpoint, the optimal cutoff was 5936.40 pg/mL, and Kaplan−Meier analysis revealed that patients in the high plasma sADAM10 group had a significantly higher risk of post−transplant infection (P = 0.028; **Figure 4A**). To assess the continuous association and avoid cut−off bias, we performed restricted cubic spline Cox regression with the 5^th^ percentile (2574.5 pg/mL) as reference, adjusting for age and sex (**Figure 4B**). This analysis confirmed a significant non−linear relationship (P for non−linearity = 0.029; **Table 2**), with plasma sADAM10 between 3704.3 and 6126.5 pg/mL associated with a significantly elevated infection risk (lower bound of 95% CI >1). Within this interval, the hazard ratio increased progressively, peaking at 4706.6 pg/mL (HR = 5.15, 95% CI: 1.54–17.23), and declined thereafter, with the confidence interval crossing unity above 6126.5 pg/mL. Notably, the previously identified optimal cut−off of 5936.4 pg/mL fell within this significant interval, further validating its clinical relevance. We further assessed whether plasma sADAM10 improves infection risk prediction beyond traditional risk factors. A baseline Cox model containing age and eGFR yielded a C−index of 0.795 (95% CI: 0.71–0.88). Adding plasma sADAM10 as a restricted cubic spline term significantly improved model fit (likelihood ratio χ² = 10.64, df = 2, P = 0.005) and increased the C−index to 0.842 (95% CI: 0.77–0.91), indicating that plasma sADAM10 provides incremental prognostic information for infection risk stratification.

**Table 2:** Restricted cubic spline Cox regression analyses of plasma sADAM10 for predicting infection and composite renal outcomes.

Endpoint	Model	Adjustment variables	Non-linearity P	Overall P
Infection	1	Unadjusted	0.031	0.096
	2	Age, sex	0.029	0.089
	3	Age, eGFR	0.064	0.163
	4	Age	0.036	0.111
Composite renal	1	Unadjusted	0.116	0.125
	2	Age, sex, eGFR, proteinuria	0.338	0.606
	3	Age, sex, eGFR, proteinuria, neutrophil count	0.356	0.653

All models used a restricted cubic spline with three knots for plasma sADAM10, with the 5th percentile as reference. Infection endpoint analyses were performed in the KTR cohort (n=105, 21 events). Composite renal endpoint (graft failure or ≥30% eGFR decline) was analyzed in the KTR cohort (n=99, 50 events).Non-linearity P refers to the Wald test for the non-linear component of the spline; Overall P refers to the joint test of the linear and non-linear components. Proteinuria (Upro) was included as a semi−quantitative variable.

For the composite renal endpoint, the optimal cutoff was 3875.1 pg/mL, and the high plasma sADAM10 group showed a substantially elevated risk in unadjusted analysis (HR = 3.37, 95% CI: 1.34–8.49, P = 0.006; **Supplementary Figure 3**). However, after adjusting for age, sex, eGFR, and proteinuria in the restricted cubic spline model, plasma sADAM10 no longer demonstrated a significant association with renal outcomes (overall Wald P = 0.606, P for non−linearity = 0.338). Adding plasma sADAM10 as a linear term to a baseline model containing these established risk factors did not improve model fit (likelihood ratio P = 0.768) and left the C−index virtually unchanged (from 0.711 to 0.715). Similarly, incorporating plasma sADAM10 as a restricted cubic spline term yielded no improvement in model fit (likelihood ratio P = 0.598) or discrimination (C−index = 0.715). These findings indicate that the apparent prognostic value of plasma sADAM10 for renal endpoints was largely confounded by renal function.

Given the limited follow-up duration in the native CKD cohort, which precluded accrual of sufficient clinical endpoint events, we utilized an established kidney failure prediction model (31) to evaluate the association between plasma sADAM10 and 5-year kidney failure risk in patients with advanced CKD (G3–G5, n = 33). An exponential relationship was identified, described by the equation: Risk = 0.1034 × exp(0.0009 × plasma sADAM10) (**Supplementary Figure 4**). This model indicates that the predicted kidney failure risk doubles with approximately every 745 pg/mL increase in plasma sADAM10 concentration, underscoring its potential for dynamic risk stratification in this population.

## 4 Discussion

The development of novel non-invasive biomarkers is critical for improving the management of KTRs and patients with CKDs, offering potential for early intervention and refined prognostication. In this study, we identified plasma sADAM10 as a context-dependent prognostic biomarker in kidney diseases. Our data demonstrated that plasma sADAM10 were significantly elevated in KTR with allograft dysfunction and in CKD patients compared with healthy controls. Multivariate analyses revealed distinct cohort-specific associations: plasma sADAM10 independently correlated with eGFR in CKD patients, whereas it was independently associated with neutrophil count in KTR, suggesting that its biological drivers differ between these populations. Furthermore, elevated plasma sADAM10 were significantly associated with the severity of key histopathological lesions, particularly interstitial fibrosis and tubular atrophy, tubular injury, and interstitial inflammation. These findings collectively indicate that plasma sADAM10 is independently associated with both the degree of renal impairment and underlying fibro-inflammatory damage, and ultimately predicts adverse outcomes including post-transplant infection.

Although post-transplant time may influence immune status and the risk of complications, the lack of a significant correlation between plasma sADAM10 and post-transplant days in this study suggests that plasma sADAM10 may primarily reflect renal pathological status rather than being a simple time-dependent change. Future studies should validate this finding through stratified analyses across different post-transplant stages. The correlation between plasma sADAM10, renal function, and pathological injury points to its potential involvement in the pathogenic cycle of inflammation and fibrosis in CKD and allograft dysfunction. Specifically, plasma sADAM10 were positively associated with neutrophil count, neutrophil-to-lymphocyte ratio, and interstitial inflammation, indicating a link to both systemic and local inflammatory processes. Given the functional synergy and co-regulation between ADAM10 and ADAM17, a key regulator of neutrophil function (32), elevated plasma sADAM10 likely reflects broad protease activation within the inflammatory renal milieu. This inflammation-driven protease activity in turn impacts renal structure, as evidenced by the correlations between plasma sADAM10 and the severity of IFTA and tubular epithelial cell injury. Previous studies have reported that ADAM10 cleaves and releases soluble forms of inflammatory mediators such as CXCL16, promoting immune cell recruitment and amplifying renal inflammation, thereby exacerbating fibrosis and injury severity (14). The more pronounced plasma sADAM10 elevation and broader histopathological correlations observed in KTR may reflect intensified immune activation following transplantation. In native CKD patients (combining MN and IgAN), the association between plasma sADAM10 and arteriolar hyalinosis suggests a potential link to hypertension or metabolic−related microvascular injury. Detailed subgroup comparisons between MN and IgAN are presented in **Supplementary Figure 2**. It should be noted that the initial correlation analyses in this study were exploratory and unadjusted for confounders. Although we subsequently performed multivariate regression models adjusting for age, sex, BMI, proteinuria, and the mutual confounding between renal function and inflammatory markers, residual confounding including unmeasured immune status and medication effects cannot be entirely excluded. Therefore, these findings should be interpreted with caution, and their independence and causal relationships warrant further validation in prospective cohorts and mechanistic studies.

An interesting finding of this study is the nonlinear relationship between plasma sADAM10 and eGFR. A significant negative correlation was observed at plasma sADAM10 below 6000 pg/mL, beyond which the association plateaued. The apparent normalization of plasma sADAM10 in stage G5 CKD does not indicate improved renal health but likely represents an end-stage phenomenon wherein biomarker production becomes limited. We propose two possible explanations for this observation. First, the profound uremic milieu in advanced CKD may alter non-renal clearance mechanisms for proteins such as sADAM10. Second, and more compellingly, the depletion of primary renal source cells including tubular epithelial cells and their substrates such as E-cadherin and Klotho due to extensive fibrosis may lead to substrate exhaustion (33). Collectively, kidney inflammation promotes ADAM10 activation, which in turn exacerbates inflammation, causes direct cell injury, and initiates fibrosis, driving progressive renal functional decline. These mechanistic insights not only offer a plausible explanation for the patterns observed in our correlation and nonlinear analyses but also suggest that plasma sADAM10 may act not only as a biomarker of deteriorating renal function but also as a potential mediator in disease progression.

This study identifies plasma sADAM10 as a context-dependent prognostic biomarker in kidney diseases. Using both cut-off-based survival analyses and continuous restricted cubic spline Cox regression, we demonstrated that plasma sADAM10 predicts infection risk in KTR with a significant non-linear relationship. The RCS analysis confirmed that the previously identified cut-off (5936.4 pg/mL) falls within the range where infection risk is significantly elevated, validating its clinical relevance while avoiding cut-off optimization bias. Notably, plasma sADAM10 exhibited an inverted U-shaped association with infection risk, such that moderate elevations may reflect a pro-inflammatory state predisposing to infection, whereas extreme elevations could represent an exhaustion phenomenon or altered clearance mechanisms. The incremental value analysis confirmed that plasma sADAM10 significantly improves infection risk prediction beyond age and eGFR, supporting its potential clinical utility in KTR. In contrast, after adjusting for eGFR and other confounders, plasma sADAM10 showed no independent association with the composite renal endpoint, suggesting that its apparent prognostic value for renal outcomes was largely confounded by baseline renal function and does not provide additional information beyond established markers. Plasma sADAM10 did not demonstrate incremental value after accounting for renal function and proteinuria, consistent with our adjusted RCS findings. These findings underscore the endpoint-specific nature of plasma sADAM10, and its utility in infection risk stratification likely reflects its role in inflammatory processes. Furthermore, in an exploratory analysis of advanced CKD patients, plasma sADAM10 demonstrated a quantitative relationship with predicted 5-year kidney failure risk (a 745 pg/mL increment associated with a two-fold risk increase), supporting its potential as a dynamic monitoring tool in non-transplant CKD, where its relationship with renal outcomes may differ from that in KTR. It is worth noting that this analysis is exploratory and hypothesis−generating. The association between plasma sADAM10 and the predicted 5−year kidney failure risk should be interpreted with caution and requires validation in larger cohorts with actual observed outcomes.

Several non-mutually exclusive mechanisms may explain elevated plasma sADAM10 in our cohorts. First, renal parenchymal injury likely contributes, as the significant association we observed between plasma sADAM10 and the severity of tubular injury and interstitial fibrosis is consistent with evidence that injured tubular epithelial cells and podocytes release ADAM10 or its cleavage products (34). Second, immune cell activation may also represent an important source. Our finding that plasma sADAM10 correlates with neutrophil count and NLR in KTR, even after adjusting for eGFR, supports this notion, as activated leukocytes upregulate ADAM10 expression and shedding activity. We previously reported that decreased monocytic ADAM10 expression was associated with allograft dysfunction, suggesting that alterations in immune cell ADAM10 dynamics may contribute to circulating sADAM10 levels (26). Notably, studies in Alzheimer’s disease have shown that plasma sADAM10 is higher in patients, particularly among APOEϵ4 carriers, suggesting potential links between genetic risk and ADAM10 shedding (28). Third, impaired renal clearance may contribute in advanced disease. The nonlinear relationship between plasma sADAM10 and eGFR, with levels plateauing in stage G5 CKD, could reflect altered clearance of middle-molecular-weight proteins. Klotho, an anti-aging protein primarily produced in the kidney, undergoes ADAM10-mediated shedding, and its soluble form is similarly influenced by renal function (35). Reduced renal clearance of uremic solutes may prolong sADAM10 half-life, analogous to asymmetric dimethylarginine accumulation in CKD (36, 37). Moreover, plasma and cerebrospinal fluid sADAM10 is predominantly inactive, suggesting that accumulation may reflect impaired clearance rather than functional upregulation (27, 38). Fourth, tetraspanin-mediated regulation may modulate ADAM10 activity and substrate specificity. Rosenbaum et al. identified a tricomponent complex of THSD7A, ADAM10, and Tspan15 in podocytes, with THSD7A acting as both an ADAM10 substrate and a stabilizer of the mature protease (17). Perturbations in this complex could influence sADAM10 levels. Similarly, Tspan5 has been implicated in CaSR-mediated Klotho shedding, further highlighting the importance of tetraspanin scaffolds in regulating ADAM10 function (35). CKD-related systemic inflammation and endothelial injury may further enhance shedding. Elevated high-sensitivity C-reactive protein can destabilize endothelial nitric oxide synthase and indirectly promote ADAM10 release (36, 39). Endothelial injury triggers release of von Willebrand factor and its propeptide, markers of endothelial activation that correlate with renal dysfunction (40). Given ADAM10’s role in cleaving endothelial adhesion molecules such as N-cadherin, its shedding may be exacerbated by endothelial stress responses, further elevating plasma levels (36, 41). Integrating these findings, we hypothesize that elevated plasma sADAM10 reflects increased production from activated immune cells and injured renal parenchyma, coupled with altered clearance in advanced stages. The relative contributions likely vary by disease context, being inflammation-driven in KTR and parenchymal damage-driven in CKD, which may explain the distinct association patterns observed between our two cohorts.

This study has several limitations that should be considered when interpreting the findings. The single-center observational design may limit the generalizability of our results, and multi-center validation is warranted. Our KTR cohort was enriched for patients undergoing indication biopsies, meaning that the findings may not be fully applicable to stable, unselected KTR, and further studies in broader populations are needed to confirm the prognostic value of plasma sADAM10 across the full spectrum of transplant recipients. The cross-sectional nature of our data precludes establishing causal relationships between plasma sADAM10 and disease progression, highlighting the need for well-designed mechanistic experiments. Additionally, the limited number of endpoint events in certain subgroups may have reduced the statistical power of our survival analyses, and the robustness of these findings requires confirmation in larger prospective studies with longer follow-up. The stable subgroup (n=31) had too few outcome events to permit any meaningful prognostic analysis, which precluded a sensitivity analysis restricted to stable patients. In particular, the restricted cubic spline analysis for infection risk was based on only 21 events, which likely explains the wide confidence intervals and high hazard ratio estimates; this finding should therefore be considered exploratory. In the KTR cohort, we adjusted for tacrolimus trough levels, which were routinely monitored. We did not have data on mycophenolate mofetil or corticosteroid concentrations or precise dose histories at the time of sampling, and therefore could not adjust for these agents. Although immunosuppressive regimens followed KDIGO guidelines, residual confounding cannot be excluded. Beyond these considerations, further mechanistic investigations incorporating cellular origin tracing, kinetic clearance studies, proteomics, and urinary sADAM10 measurements will be essential to elucidate the precise sources and pathophysiological role of circulating sADAM10. Such work may ultimately inform therapeutic strategies targeting ADAM10 shedding or clearance pathways in both neurodegenerative and renal diseases.

## 5 Conclusion

This study establishes plasma sADAM10 as a context-dependent biomarker with endpoint-specific prognostic value in kidney diseases. Through systematic analysis of healthy individuals, CKD patients, and KTR, we demonstrate that plasma sADAM10 is independently associated with renal dysfunction in CKD and inflammatory activation in KTR. Plasma sADAM10 exhibits a significant non-linear association with post-transplant infection risk and provides incremental prognostic value beyond traditional markers such as age and eGFR. In contrast, its apparent association with renal outcomes is largely confounded by baseline renal function, and it does not offer independent prognostic information for the composite renal endpoint after adjusting for eGFR. In advanced CKD patients, plasma sADAM10 shows a quantitative relationship with predicted kidney failure risk, supporting further exploration in non-transplant settings. Future efforts should focus on validating its clinical utility in large, multi-center cohorts and elucidating the molecular mechanisms governing its production and function, particularly its role in inflammation and infection risk.

## Data Availability

The raw data supporting the conclusions of this article will be made available by the authors, without undue reservation.
