# Supplementary material for: Clinical implications of plasma sADAM10: integrating renal function, histopathology and prognostic outcomes in chronic kidney disease and kidney transplantation
Source: Front Immunol. 2026 Jul 2;17:1752330. doi: 10.3389/fimmu.2026.1752330 (PMC13372577; doi:10.3389/fimmu.2026.1752330)
Supplement: Supplementary file 1 [file DataSheet1.docx]

Supplementary Material

# Supplementary Figure


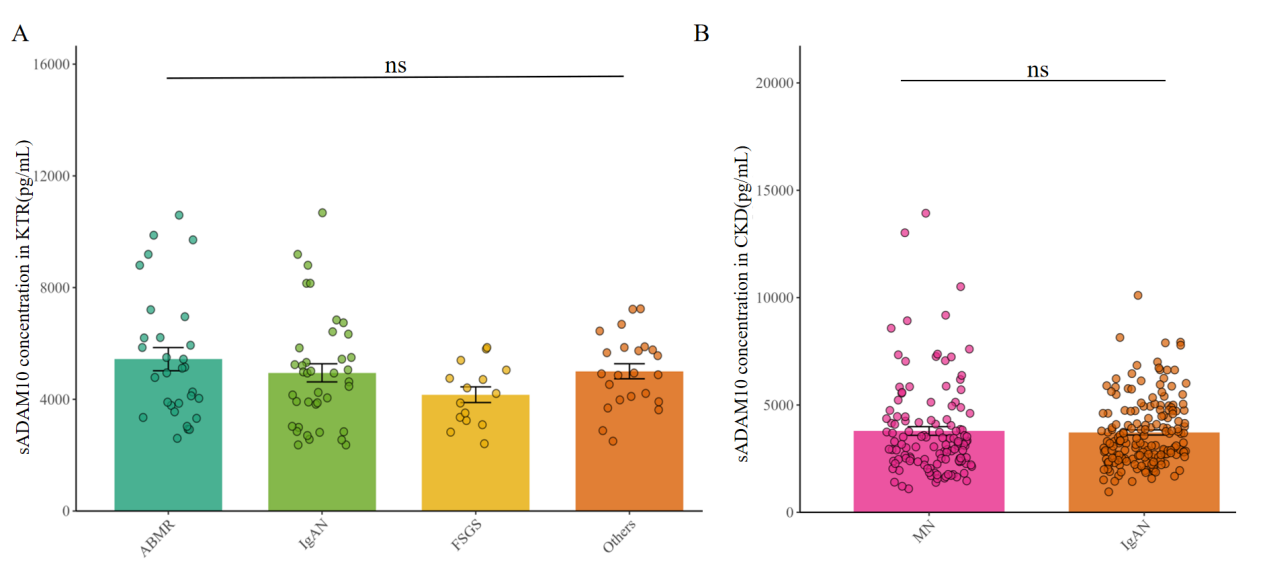


**Supplementary Figure 1** Comparison of plasma sADAM10 levels among different pathological groups in KTR (A) and patients with CKD (B)


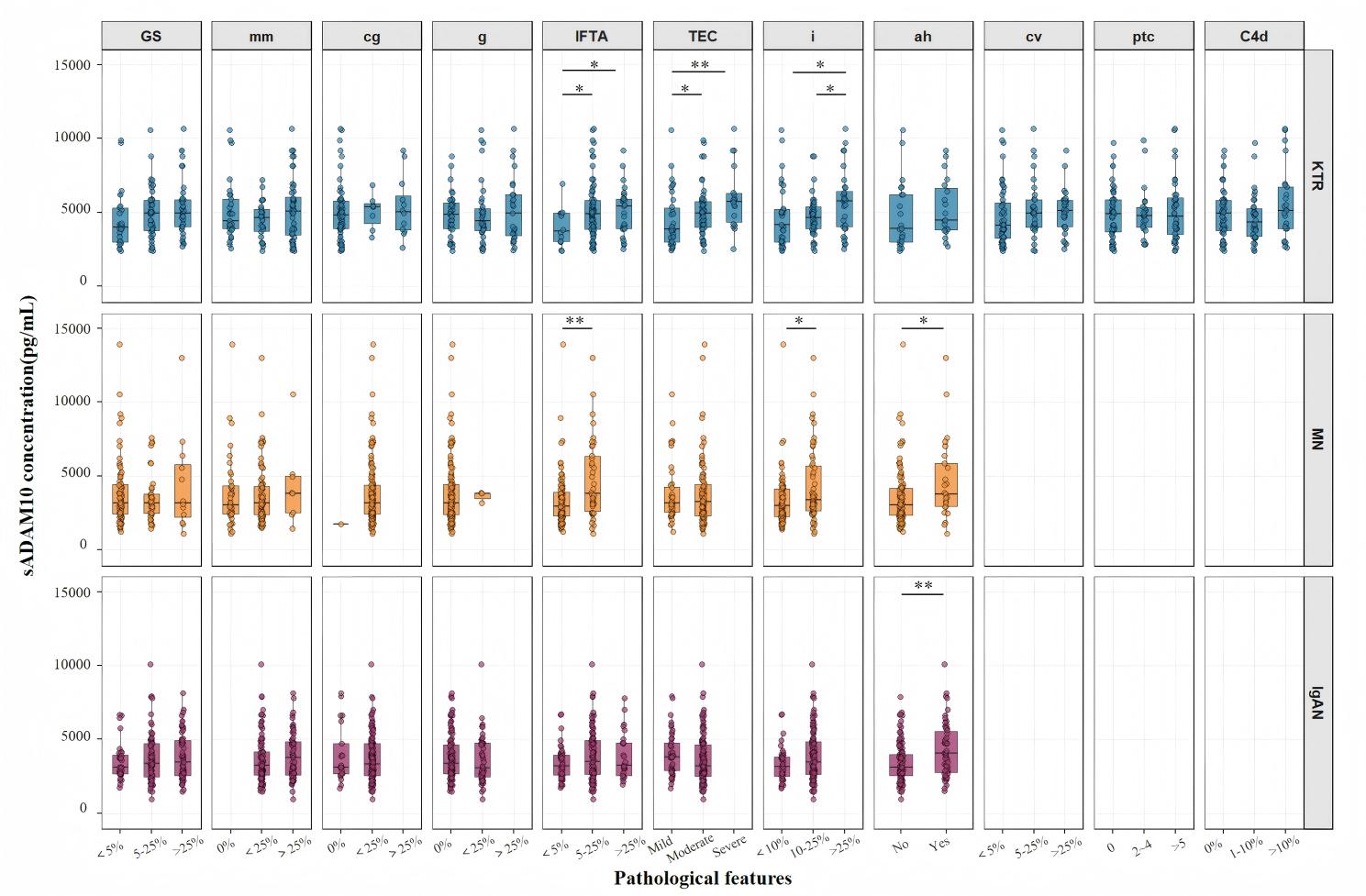


**Supplementary Figure 2** Analysis of plasma sADAM10 concentration and pathological parameters in KTR and CKD patients (detailed subgroup comparisons between MN and IgAN)

Notes: *, P<0.05; **, P<0.01; GS, glomerulosclerosis (% of glomeruli); mm, mesangial matrix expansion (% of glomerular involvement); cg, chronic glomerulopathy (% of glomerular scarring); g, glomerulitis (% of glomerular involvement); IFTA, interstitial fibrosis and tubular atrophy (% of cortical area); i, interstitial inflammation (% of cortical area); cv, vascular fibrous intimal thickening (% of luminal stenosis); C4d, C4d staining (% of peritubular capillary positivity); TEC, tubular epithelial cell injury (graded as: mild, moderate, severe); ah, arteriolar hyalinosis (graded as: present or absent); ptc, peritubular capillaritis (graded as: 0, 2-4, or >5 inflammatory cells per tubule).


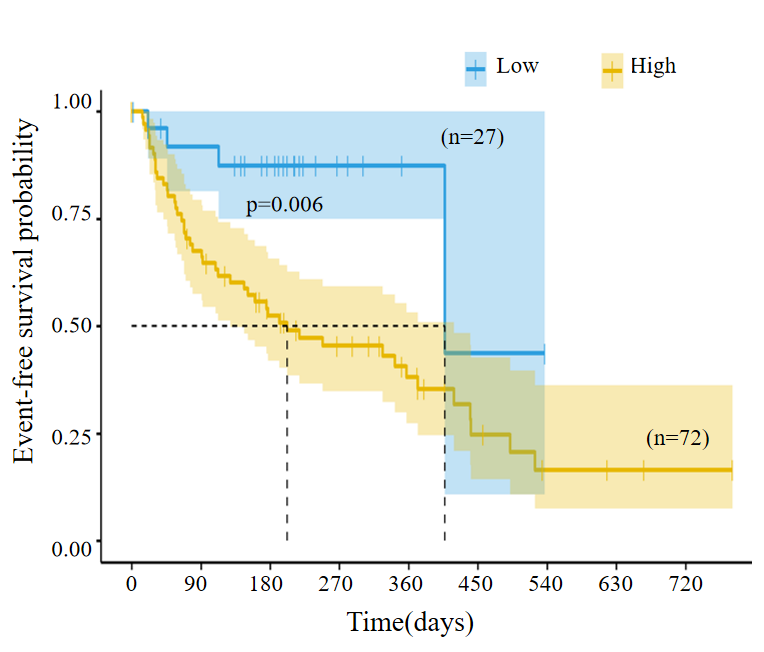


**Supplementary Figure 3** Kaplan-Meier analysis for composite renal outcomes in the KTR


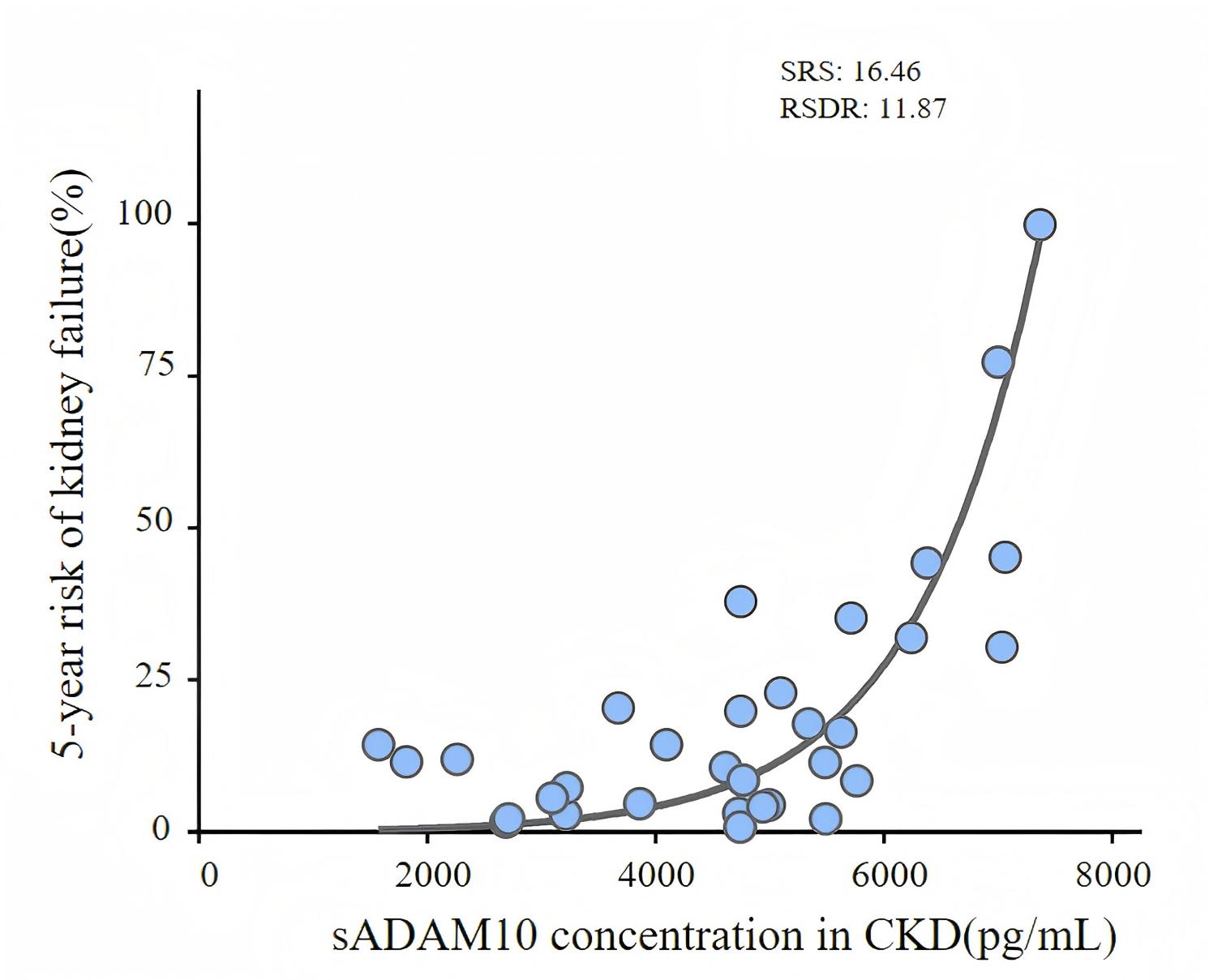


**Supplementary Figure 4** Exploratory analysis of the association between plasma sADAM10 and the predicted 5-year risk of kidney failure in patients with advanced CKD

Notes: Scatter plot showing the relationship between plasma sADAM10 levels and the predicted 5-year risk of kidney failure, calculated using the Tangri equation, in patients with advanced chronic kidney disease (CKD). An exponential curve is fitted to the data for hypothesis‑generating purposes. This exploratory analysis suggests a potential nonlinear association that warrants further validation.

**Supplementary Table 1 Baseline characteristics of kidney transplant recipients with stable allograft function versus indication biopsy.**

| Variable | Stable (n=31) | Indication-biopsy (n=114) | *P* value |
| --- | --- | --- | --- |
| Age, years | 37.0 (31.5–47.0) | 37.0 (31.0–45.8) | 0.761 |
| Male sex, n (%) | 16 (51.6) | 90 (78.9) | 0.005 |
| BMI, kg/m² | 20.0 (18.1–23.5) | 21.4 (19.6–25.0) | 0.188 |
| Time post‑transplant, days | 1828 (1224–2630) | 1541 (782–2392) | 0.408 |
| sADAM10, pg/mL | 3152 (2765–3787) | 4811 (3640–5830) | <0.001 |
| eGFR, mL/min/1.73m² | 78.2 (75.8–84.7) | 36.8 (22.0–53.2) | <0.001 |
| Serum creatinine, μmol/L | 89 (82–102) | 193 (135–274) | <0.001 |
| Urea, mmol/L | 6.4 (5.3–6.9) | 11.2 (8.5–18.1) | <0.001 |
| Cystatin C, mg/L | 1.14 (1.04–1.25) | 2.20 (1.68–3.15) | <0.001 |
| UACR, mg/g | 31.8 (23.6–39.9) | 1297 (962–3513) | 0.024 |
| Urine protein dipstick, n (%) |  |  | <0.001 |
| – | 21 (67.7) | 14 (12.3) |  |
| ± | 6 (19.4) | 8 (7.0) |  |
| 1+ | 2 (6.5) | 26 (22.8) |  |
| 2+ | 1 (3.2) | 40 (35.1) |  |
| 3+ | 1 (3.2) | 20 (17.5) |  |
| 4+ | 0 (0.0) | 6 (5.3) |  |
| Albumin, g/L | 45.8 (43.6–47.5) | 40.9 (37.0–43.4) | <0.001 |
| Triglycerides, mmol/L | 1.29 (0.88–1.69) | 1.81 (1.25–2.35) | 0.001 |
| Hemoglobin, g/L | 137 (123–151) | 123 (104–143) | 0.003 |
| Neutrophils, ×10⁹/L | 4.30 (3.37–5.50) | 6.02 (4.70–7.84) | <0.001 |
| Lymphocytes, ×10⁹/L | 2.21 (1.87–2.66) | 1.48 (0.78–2.24) | <0.001 |
| Tacrolimus level, ng/mL | 5.28 (4.44–6.06) | 5.42 (4.58–6.81) | 0.373 |

Notes: Data are presented as median (IQR) or n (%). *P* values from Mann–Whitney U test (continuous) or Fisher’s exact test (categorical).

**Supplementary Table 2 Multivariate linear regression analyses of factors associated with plasma sADAM10 levels in CKD and KTR cohorts.**

| Cohort | Parameter | Model | Adjustment variables | β(95% CI) | P value | Standardized β (Model 4) |
| --- | --- | --- | --- | --- | --- | --- |
| CKD | eGFR | 1 | Unadjusted | -0.005 (-0.007, -0.003) | <0.001 |  |
|  |  | 2 | + age, sex, BMI | -0.004(-0.006, -0.002) | <0.001 |  |
|  |  | 3 | + proteinuria | -0.004 (-0.006, -0.002) | <0.001 |  |
|  |  | 4 | + neutrophil count | -0.004 (-0.006, -0.002) | <0.001 | -0.237 |
|  | Neutrophil | 1 | Unadjusted | 0.006 (0.001, 0.011) | 0.022 |  |
|  |  | 2 | + age, sex, BMI | 0.004(-0.001, 0.010) | 0.081 |  |
|  |  | 3 | + proteinuria | 0.005 (-0.000, 0.001) | 0.072 |  |
|  |  | 4 | + eGFR | 0.003 (-0.002, 0.008) | 0.222 | 0.07 |
|  | NLR | 1 | Unadjusted | 0.028 (0.006, 0.050) | 0.013 |  |
|  |  | 2 | + age, sex, BMI | 0.021 (-0.001, 0.043) | 0.062 |  |
|  |  | 3 | + proteinuria | 0.022(-0.000, 0.044) | 0.054 |  |
|  |  | 4 | + eGFR + neutrophil | 0.022 (-0.015, 0.060) | 0.241 | 0.116 |
| KTR | eGFR | 1 | Unadjusted | -0.002 (-0.004, 0.000) | 0.075 |  |
|  |  | 2 | + age, sex, BMI | -0.002 (-0.005, 0.000) | 0.103 |  |
|  |  | 3 | + proteinuria | -0.002 (-0.005, 0.001) | 0.116 |  |
|  |  | 4 | + neutrophil count | -0.001 (-0.004, 0.002) | 0.567 | -0.067 |
|  | Neutrophil | 1 | Unadjusted | 0.009 (0.003, 0.015) | 0.002 |  |
|  |  | 2 | + age, sex, BMI | 0.010 (0.003, 0.017) | 0.009 |  |
|  |  | 3 | + proteinuria | 0.010 (0.002, 0.017) | 0.010 |  |
|  |  | 4 | + eGFR | 0.009 (0.001, 0.017) | 0.034 | 0.237 |
|  | NLR | 1 | Unadjusted | 0.010 (-0.000, 0.020) | 0.058 |  |
|  |  | 2 | + age, sex, BMI | 0.006 (-0.006, 0.018) | 0.339 |  |
|  |  | 3 | + proteinuria | 0.006 (-0.007, 0.018) | 0.355 |  |
|  |  | 4 | + eGFR + neutrophil | -0.018 (-0.037, 0.002) | 0.074 | -0.289 |

Notes: β represents the change in log-transformed plasma sADAM10 per unit increase in the predictor. Standardized β is shown only for the fully adjusted model (Model 4). Abbreviations: CKD, chronic kidney disease; KTR, kidney transplant recipient; eGFR, estimated glomerular filtration rate; NLR, neutrophil-to-lymphocyte ratio; BMI, body mass index.
